# Mitigation of artifacts in imaging biosamples with optical scanning transmission electron microscopy

**DOI:** 10.1186/s42649-026-00132-y

**Published:** 2026-04-21

**Authors:** Arent J. Kievits, B. H. Peter Duinkerken, Ryan Lane, Ben N. G. Giepmans, Jacob P. Hoogenboom

**Affiliations:** 1 Department of Imaging Physics, Delft University of Technology, Delft, The Netherlands; 2 Department of Biomedical Sciences, University Medical Center Groningen, University of Groningen, Groningen, The Netherlands

**Keywords:** Electron microscopy, Artifacts, OSTEM, Scintillators, Large-scale electron microscopy

## Abstract

Electron microscopy (EM) allows ultrastructural analysis of biological tissues and cells, but images frequently contain artifacts because biological samples have to undergo rigorous preparation to be resistant to vacuum conditions and electron beam exposure. Knowledge about the appearance of image artifacts and how they arise is crucial for their recognition and mitigation and for proper image interpretation. How artifacts appear depends strongly on the electron detection modality and the imaging conditions. Optical scanning transmission EM (OSTEM) is a detection technique compatible with single-beam and multibeam electron microscopes, in which tissue samples are directly deposited on a scintillator for imaging in transmission mode. Here, we identified several types of artifacts that may occur in single-beam and multibeam OSTEM. These artifacts arise or appear as a result of combining established sample preparation protocols with solid scintillator substrates and optical transmission detection. Artifacts can be effectively mitigated or minimized to ultimately enable high quality large-scale 2D and 3D acquisitions.

**Supplementary Information:**

The online version contains supplementary material available at 10.1186/s42649-026-00132-y.

## Introduction

Artifacts are common in electron microscopy (EM) (Maunsbach and Afzelius 1998). The sample has to meet several requirements for successful imaging with an electron beam, such as resistivity to high vacuum conditions and electron radiation damage. Artifacts complicate image interpretation, as they may alter or obscure the native structure of the sample. Thus it is crucial to recognize and understand the origin of artifacts, in order to prevent them or limit their effect on interpretation.

A very common cause of artifacts is sample charging, leading to image contrast differences and distortions. Negative charging may induce strong local fields and eventually lead to electric breakdown, which can damage the sample (Cazaux 1986). How these charging artifacts appear in the image is further influenced by the sample geometry, tilt angle and used imaging conditions, which affect the total electron yield from the sample (Reimer 2000). Irradiation by the electron beam heats the sample and may cause it to melt, shrink, deform or thermally decompose. Most radiation damage is caused by ionisation (carbon bond-breaking), resulting in the formation of carbon double bonds in organic samples (Reimer 2000). Finally, contrast differences can be created through contamination by hydrocarbon molecules on the specimen surface.

Sample charging, damaging and contamination in EM have been intensively investigated. Strategies for optimizing imaging conditions to control, mitigate or prevent these artifacts altogether have been developed accordingly, such as heavy metal staining or charge compensation with N2 flow over the sample in the chamber (Joy and Joy 1996; Höche et al. 2006; Titze 2013; Egerton 2013; Bouwer et al. 2016; Xu et al. 2017; Deerinck et al. 2018; Hugenschmidt et al. 2023). Additionally, exquisite preparation protocols have been developed to preserve the natural structure of the sample as best as possible, but some of these biological sample preparation protocols generate artifacts, which in turn have been extensively documented as well (Crang and Klomparens 1988; Hoetelmans et al. 2001). Conventionally, an electron microscopist would distinguish known artifacts from structures native to the specimen, and circumvent them by imaging an artifact-free region in the sample. Modern EM techniques commonly known as large-scale EM (Sokol et al. 2015) and volume EM (vEM, Peddie et al. (2022)) allow imaging of large areas or volumes, providing an unbiased view of the sample. Because these techniques typically produce datasets of large areas or entire sections, artifacts will inevitably be included in the acquisition. How and whether artifacts appear in the datasets is ultimately determined by the type of electron microscope, the mode of operation and the detection scheme.

The development of multibeam scanning EM (Eberle et al. 2015; Ren and Kruit 2016), which increases acquisition speed by scanning the sample with multiple beams in parallel, has motivated the use of optical tranmission detection (OSTEM) (Ren and Kruit 2016; Zuidema and Kruit 2020; Kievits et al. 2024). In OSTEM, a scintillator substrate separates the signals by conversion to a photon signal, as an alternative to detection schemes based on secondary electrons (Eberle et al. 2015). However, such a detection scheme may also reveal or introduce (new) artifacts. Compared to other scintillation-based detectors (e.g. Everhart-Thornley), the electron beam interaction volume with the scintillator substrate in OSTEM is tightly confined to a small region encompassing the sample-substrate interface, the coating layer and scintillator surface, which are also revealed.

Here, we report artifacts that appear as a result of combining single-beam and multibeam OSTEM with conventional biological sample preparation protocols for large-scale imaging and using scintillator-based solid substrates. These artifacts are typically absent in conventional array tomography studies that combine solid substrates with backscattered (BSD) or secondary electron (SE) detection schemes. Multibeam OSTEM can introduce specific artifacts and these can be aggravated by image processing. Lastly, procedures are presented to minimize or mitigate these artifacts in future experiments.

## Results

### Optical transmission detection visualizes artifacts

**Fig. 1:**
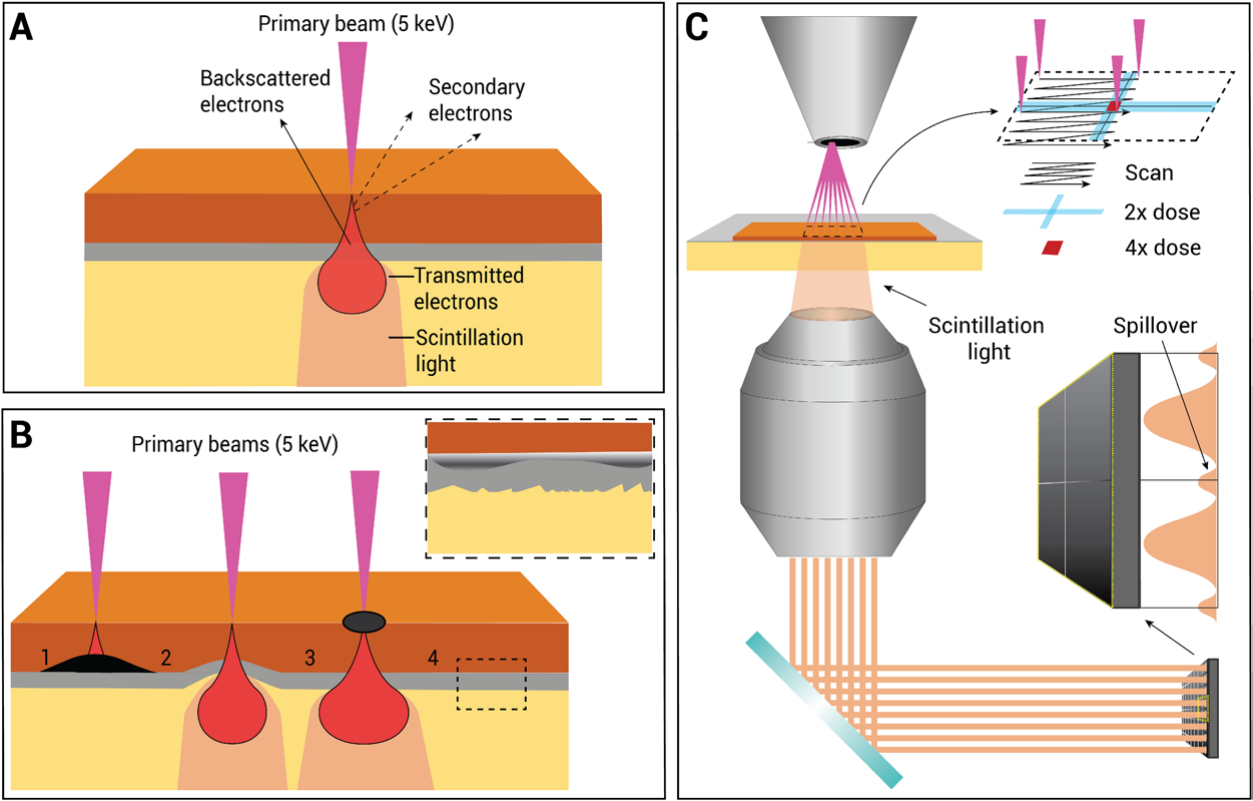
OSTEM detection scheme and associated artifacts. **A** In OSTEM, the interaction volume extends through the coating and into the scintillator, thus revealing the underlying substrate. **B** Four kinds of possible artifacts in OSTEM: (1) transmitted electrons are blocked by electron-dense features on the sample-substrate interface; (2) micro-scale elevations in the substrate profile are captured by the detection scheme; (3) increased contrast lowers spillover signal; (4) inhomogeneities in the coating layer and scintillator roughness deteriorate the image quality. **C** Multibeam OSTEM leads to additional artifacts, such as contrast differences in double or quadruple dosed areas and spillover of signal to adjacent detectors

In OSTEM, ultrathin biological sections are placed on a thin film-coated scintillator substrate, which converts transmitted electrons into photons (Fig. 1A). These photons are collected by a high NA optical objective and projected onto a multipixel photon counter. The interaction volume of the electron beam extends into the coating and scintillator, revealing any features between the substrate and sample and elevations in the substrate profile with transmission contrast (Fig. 1B). Contrast differences generated by inhomogeneities in the coating and substrate are directly superimposed on the tissue contrast. The transmission may also be modulated or blocked by electron-dense features in and on top of the sample. In multibeam OSTEM detection (like the commercial FAST-EM (Fermie et al. 2021)), additional effects must be taken into account, such as contrast differences introduced by double or quadruple dosing of overlapping scan areas and signal spillover to adjacent detectors (Fig. 1C).

### Post-staining generates artifacts on the sample-substrate boundary

**Fig. 2:**
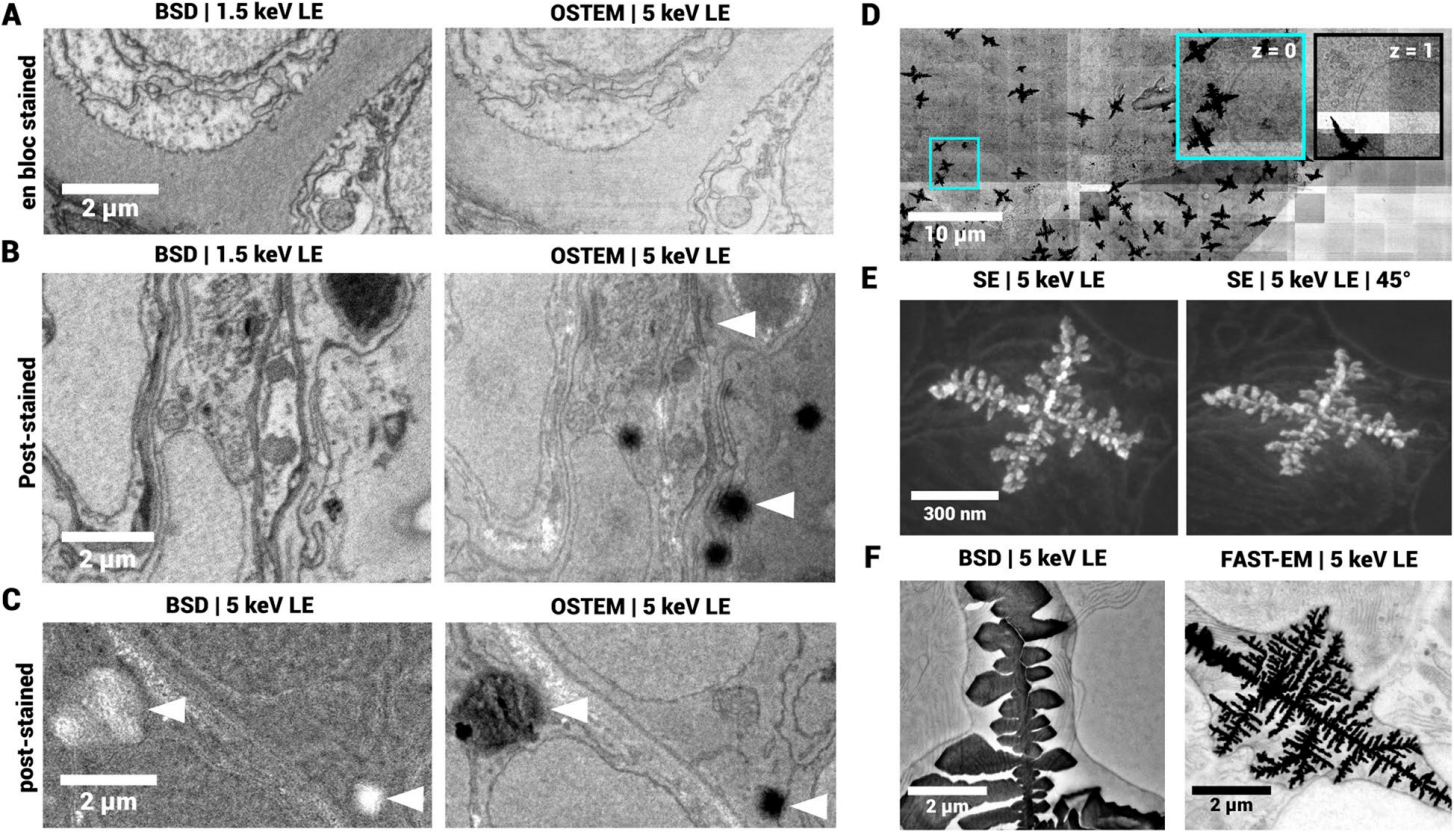
Artifacts formed during sample preparation. **A** BSD (Backscattered electron detection) and OSTEM images of 80nm thin sections of zebrafish larva *en bloc* stained with 4% neodymium acetate. **B** BSD and OSTEM images of 80nm thin sections of zebrafish larva post-stained with 4% neodymium acetate. Arrows indicate artifacts present in OSTEM image, but not in BSD image at 1.5 keV landing energy. **C** BSD with the same landing energy as OSTEM (5keV), now revealing the same artifacts (indicated by arrows), although the contrast mechanism is compromised due to the suboptimal landing energy. **D** Electron-dense precipitates in large-scale acquisitions of rat pancreas with FAST-EM (unprocessed images). Cyan inset shows endocrine tissue. The right panel shows the same cryan region, but in the adjacent serial section. **E** Single precipitate in secondary electron detection at 0 and 45 degree tilt angle. **F** Images of precipitates with backscattered electron detection and multibeam OSTEM (FAST-EM). Full dataset in **D** can be accessed at Nanotomy.org

Previously, various staining protocols were tested for compatibility with multibeam OSTEM (Duinkerken et al. 2025). *En bloc* staining provides uniform tissue contrast (Fig. 2A). Post-stained tissue sections imaged with OSTEM, as opposed to sections with *en bloc* staining, demonstrate artifacts with a darker contrast than the surrounding tissue (Fig. 2B, Duinkerken et al. (2025)). Interestingly, backscattered electron detection (BSD) at ${1.5}\,\text {keV}$ landing energy does not reveal artifacts in the post-stained sections (Fig. 2B), whereas increasing the landing energy to that of OSTEM (${5}\,\text {keV}$) does (Fig. 2C). Moreover, the artifacts are not observed outside of the section on the substrate (Data not shown), although the staining solution is applied there. Collectively, these observations suggest that the artifacts originate at the interface between the section and the substrate, rather than within the tissue section. We rationalize that these artifacts are thus created by residual staining solution that is trapped under the section by capillary force.

### Interaction between the coating layer and the sample

Molybdenum nanofilms adhere firmly to glass, have high electrical and thermal conductivity, are dimensional stable and corrosion resistant (Chauhan et al. 2022). The thin molybdenum coating of scintillators has two additional functions in OSTEM image formation. It acts as a spacer between the sample and the scintillator. Electrons traveling through the coating layer scatter further before they interact with the scintillator. Moreover, the metallic coating is reflective, a property which may aid in enhancing the signal collection by redirecting photons that are emitted towards the sample-scintillator surface back to the objective lens.

The nature and composition of the molybdenum thin film surface are affected by the medium (air, aqueous), pH and processing parameters (temperature, argon pressure and radiofrequency power) (Saji and Lee 2012; Kalaswad et al. 2023). Specific electron-dense precipitates formed in pancreas tissue prepared with reduced osmium-thiocarbohydrazide-osmium (rOTO) protocol on molybdenum-coated scintillators (Fig. 2D). The sections were taken from the knife bath using a loop and then deposited on a droplet of water placed on the scintillator. The precipitates resemble fractal-like structures which are typically created by diffusion limited aggregation, possibly caused by a reaction between the coating layer and a compound in the sample preparation protocol, mediated by the aqueous medium.

High-magnification secondary electron images clearly distinguish the precipitate structure (Fig. 2E), and complementary images of a different sample indicate that the precipitates can be located both on top of and below the section, and differ in size (Fig. 2F). In some cases, the tissue ultrastructure is obscured (Fig. 2E), but images taken with BSD show distinguishable tissue ultrastructure overlaid with and curving of the tissue section around the precipitate (Fig. 2F). In the complementary multibeam OSTEM image, the ultrastructure is obscured, indicating that the precipitates block the transmission signal.

Since the precipitates are not continuous in serial sections of the same sample (Fig. 2D), they must form after embedding, likely during section collection on the substrate. The precipitates do not form when depositing sections on scintillators with coatings other than molybdenum (e.g. chromium or graphene, data not shown). Moreover, precipitates are mostly present in tissue and only very marginally in the surrounding empty resin (Fig. 2D). Thus, a reaction with a compound in the resin seems unlikely. The precipitates do not form on the tissue sections when prepared according to the protocol for FAST-EM array tomography (Kievits et al. 2024), where the scintillator is submersed in the knife boat and then dried by lowering the water level. In short, the coating layer may interact with the sample to form artifacts, possibly mediated by water droplets on the substrate. Thus, while the coating layer may interact with the sample to form artifacts, their formation can be mitigated by submerging the substrate in the water bath of the diamond knife (Duinkerken et al. 2025).

### Coating stability affects image contrast in OSTEM

For high quality specimen preparation in array tomography, a homogeneously sputtered coating stable to prolonged water exposure is required. A low quality film may be a result of poor adhesion to the surface, which can be affected by film stress (Dai et al. 2014), surface roughness (Whitacre et al. 1998), surface contaminants (Mattox 1978) and particle contamination induced by the sputtering process (Selwyn et al. 1998). Molybdenum thin films demonstrate surface oxidation at room temperature in exposure to (moisturized) air (Founta et al. 2022; Spevack and McIntyre 1990; Stewart and Fleischauer 1982; List et al. 2009), which in turn slows down further oxidation. Furthermore, the film properties may change by a reaction with compounds in the specimen, as discussed earlier.

**Fig. 3:**
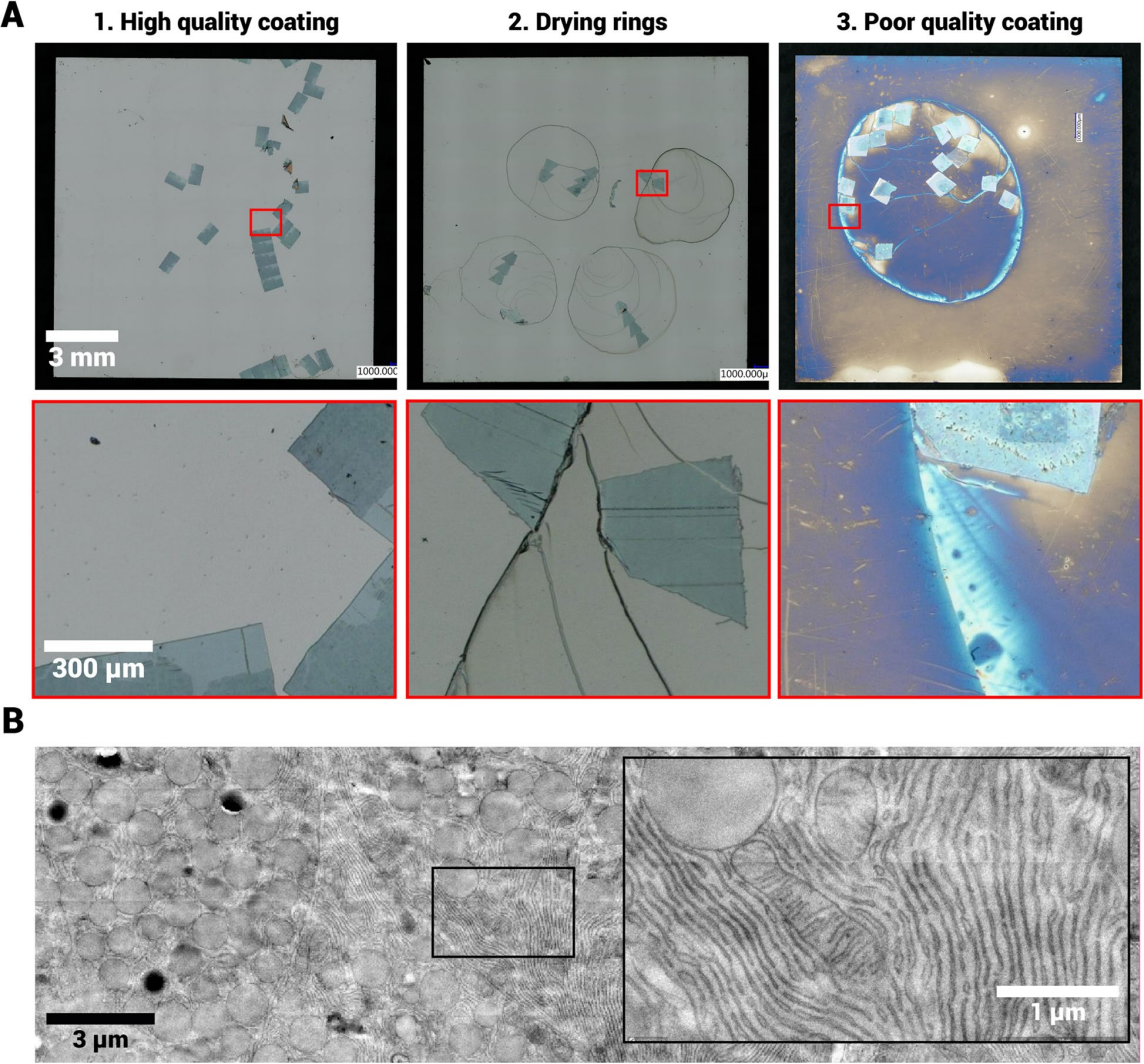
Low quality molybdenum films affect the image contrast in OSTEM. **A** 1: scintillator with unaffected coating. 2: scintillator with coating which is clearly changed by the sample preparation or exposure to air or water. 3: scintillator which shows drying patterns where the water was in contact with the coating. **B** Large-scale acquisition of section on scintillator 3 from **A**. Inset shows ultrastructure at full resolution. Dwell time: 20$\,\upmu$s. The full dataset can be accessed at Nanotomy.org

High quality sputtered molybdenum films have a homogeneous, grey reflective surface, which retains in the water during sectioning (Fig. 3A 1). However, some scintillators demonstrate drying rings (Fig. 3A 2), or become more transparent (Fig. 3A 3), possibly indicative of oxidation of the molybdenum layer in contact with air or moisture. The effect of a low quality coating on the image quality is further exemplified by images from a scintillator with demonstrated low surface roughness but an affected molybdenum film as identified by visual inspection. The contrast from the biological sample is clearly affected in FAST-EM acquisitions (Fig. 3B), although the ultrastructure remains discernible. Thus, visual inspection should precede and follow specimen preparation such that high-quality imaging is not precluded.

### Substrate surface roughness, scratches and defects deteriorate OSTEM image quality

The quality of the substrate surface finish affects the image quality in OSTEM, notably the image contrast (Zuidema 2023). Datasets exhibited apparent differences in image quality, present even in datasets from multiple specimens prepared with identical sample preparation protocols, and both in single-beam and multibeam OSTEM recordings. Therefore, we reasoned that the image quality is affected by variations in substrate quality, possibly resulting from differences between batches. To what extent the surface roughness impacts the biological contrast has not been quantified. Any surface defects in the substrate and coating comparable to or larger than the beam profile impinging on the surface will cumulatively contribute to the image formation and thereby reduce the biological contrast.

Surface scans of uncoated scintillators with atomic force microscopy (AFM) show large differences in surface roughness between batches and suppliers (Fig. S1). One scintillator (batch 1) exhibits a particularly high surface roughness, covered with numerous scratches and defects. A different scintillator from the same supplier (batch 2) shows lower surface roughness but still many apparent scratches. Scintillators from a different supplier (Surface Preparation Laboratory, “SPL”) are of higher quality, demonstrating little to no defects and near-atomic level surface roughness.

**Fig. 4:**
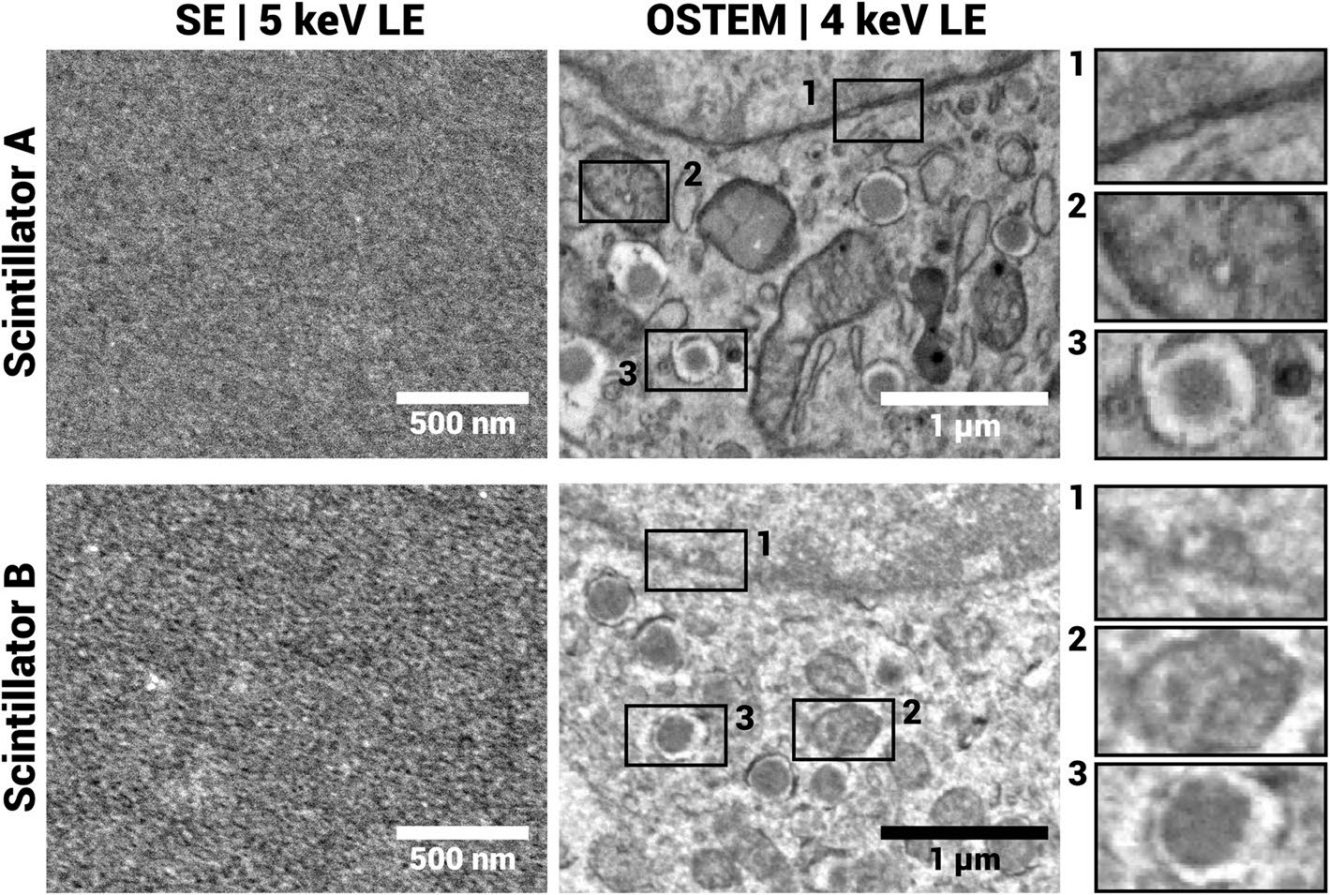
Secondary electron (SE) images of the substrate surface, and OSTEM images of biological samples, from two different scintillators (**A** and **B**). The surface SE contrast is clearly increased for **B**, which translates to reduced contrast in the biological image (OSTEM). Insets show from top to bottom: a nuclear membrane, mitochondria (supposedly) and a granule. OSTEM datasets of rat pancreas on scintillator **A** and **B** can be viewed via Nanotomy.org (**A**: https://webknossos.tnw.tudelft.nl/links/2ZPNDV1wzw56z0Xh and **B**: https://webknossos.tnw.tudelft.nl/links/eOq0IGVQx3F6wFyQ). SE images were acquired with a 5 $\upmu$ s dwell time, OSTEM images with a 10 $\upmu$ s dwell time (Scintillator **A**) and 20 $\upmu$ s dwell time (Scintillator **B**), respectively

Coated scintillators with biological samples (subsequently referenced to as “A” and “B”) exhibited notable difference in secondary electron (SE) contrast of the substrate surface (Fig. 4), likely due to a rougher surface, though the surface profile is similar (supposedly the contrast comes predominantly from the molybdenum film). The apparent biological image quality on B is compromised with respect to A, appearing more grainy. In the image from A, small features such as the nuclear membrane and mitochondrial cristae can be discerned, whereas in the image of B these features are lost.

**Fig. 5:**
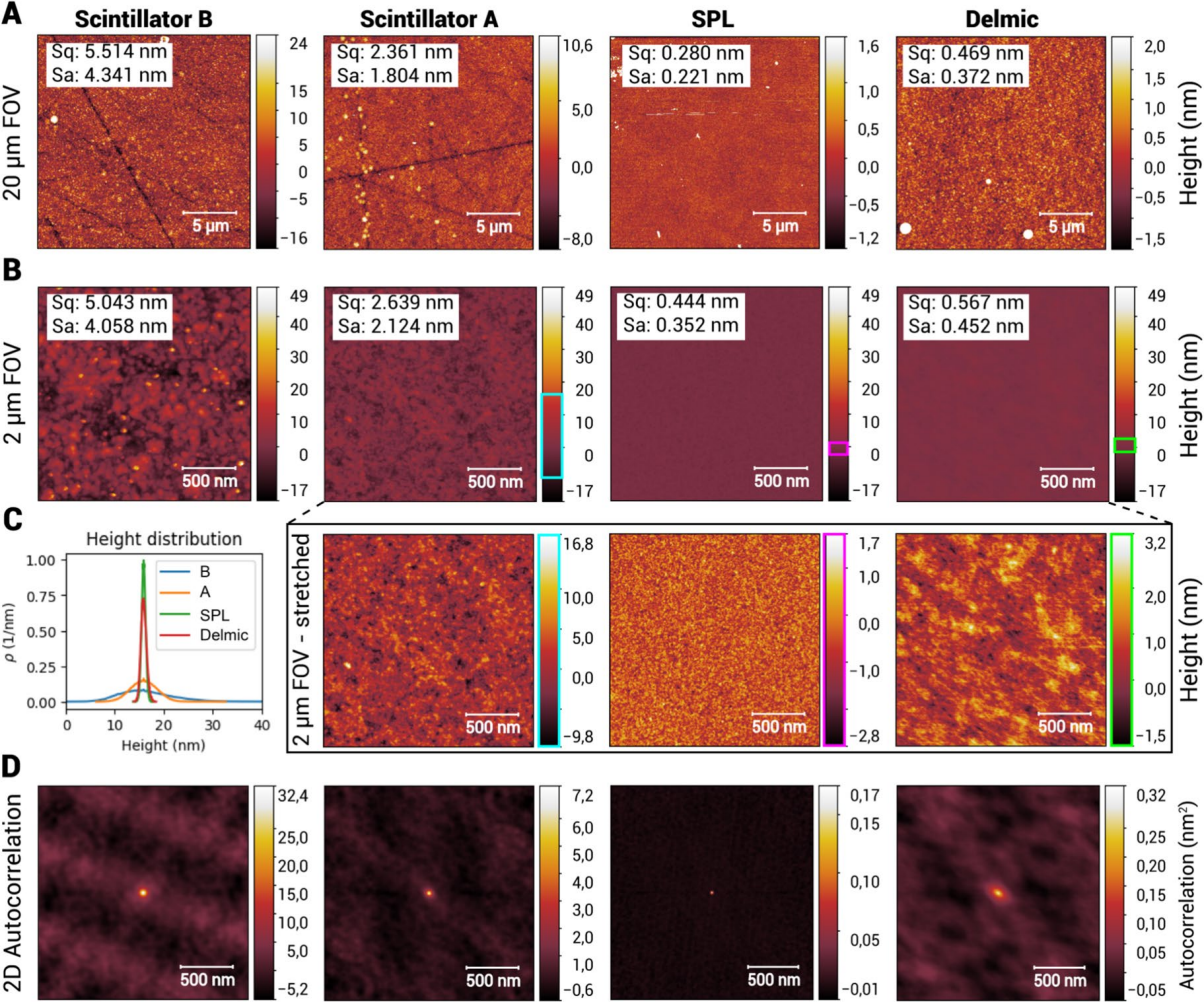
AFM measurements of molybdenum-coated YAG scintillators, showing a link between biological image quality and substrate roughness as well as an improved surface finish of supplied substrates. Scintillators were compared from different batches and suppliers, with biological specimen (A/B, Fig. 4) and without (Surface Preparation Laboratory/Delmic), as shown in the order of acquisition from left to right. Coated scintillators demonstrated varying roughness, with the SPL and Delmic scintillators having the lowest roughness. **A** 20 $\upmu$ m field-of-view; **B** 2 $\upmu$ m field-of-view, at same intensity scale. Inset shows intensities scaled to individual minimum and maximum intensities. **C** Height distribution of images in **B**, where $\rho$ is probability density of heights. **D** 2D autocorrelation of images in **B**. Images were acquired and processed as described previously. *Sq*: RMS roughness; *Sa*: mean roughness

Root-mean-square (Sq) and mean (Sa) roughness were determined by AFM for the A and B scintillators as well as two different coated scintillators from different suppliers (Surface Preparation Laboratory: “SPL” and “Delmic”, based on the supplier) that did not undergo specimen preparation (Fig. 5A,B). Prior to these experiments, the main supplier (Delmic) improved the variability in surface roughness between scintillator batches, and the “Delmic” scintillator represents the optimized substrate quality. The surface roughness varies substantially between scintillators as shown by the height distribution (Fig. 5C). Autocorrelation plots of the surface show a periodic pattern over about a micrometer length scale for scintillator B, but not for the other scintillators (Fig. 5D). The highest surface roughness is found, as expected, for the B scintillator (Sq: 5.0-5.5 nm, Sa 4.3-4.0 nm). Notably, the A scintillator has a twofold reduced surface roughness compared to B (Sq: 2.6-2.4 nm, Sa: 2.1-1.8 nm). The “SPL” and “Delmic” scintillators have the lowest surface roughness (Sq: 0.28-0.44 nm, Sa: 0.22-0.35 nm, Sq: 0.47-0.57, Sa: 0.37-0.45 respectively) and the lowest peak heights. These scintillators also do not show any scratch marks, indicating that their surface polishing is better. Scintillators from the same batch have subsequently demonstrated high biological image quality (“SPL”: see Kievits et al. (2024), “Delmic”: Fig. 7). The 2$\,\upmu$m FOV scan of the SPL scintillator (Fig. 5B, black inset) represents the texture of the molybdenum coating, as the image is apparently similar to the SE images of scintillator A (Fig. 4).

To further establish that the variation in OSTEM image quality originated from the substrate roughness and to exclude differences in sample preparation, BSD images were acquired with increasing landing energy from 1.5 to 4 keV (Fig. S2), a range of energies in which the electron beam first only probes the biological section at lower energies and then extends into the coating and scintillator. The apparent contrast and image quality is initially similar for both samples, but a more apparent degradation of contrast is observed for tissue on scintillator B as compared to A from 3 keV and higher, most notably in homogeneous regions such as cell nuclei and empty resin. Thus, the OSTEM image quality is negatively affected by irregularities in the substrate surface.

### Scintillator growth and polishing leads to characteristic artifacts

**Fig. 6:**
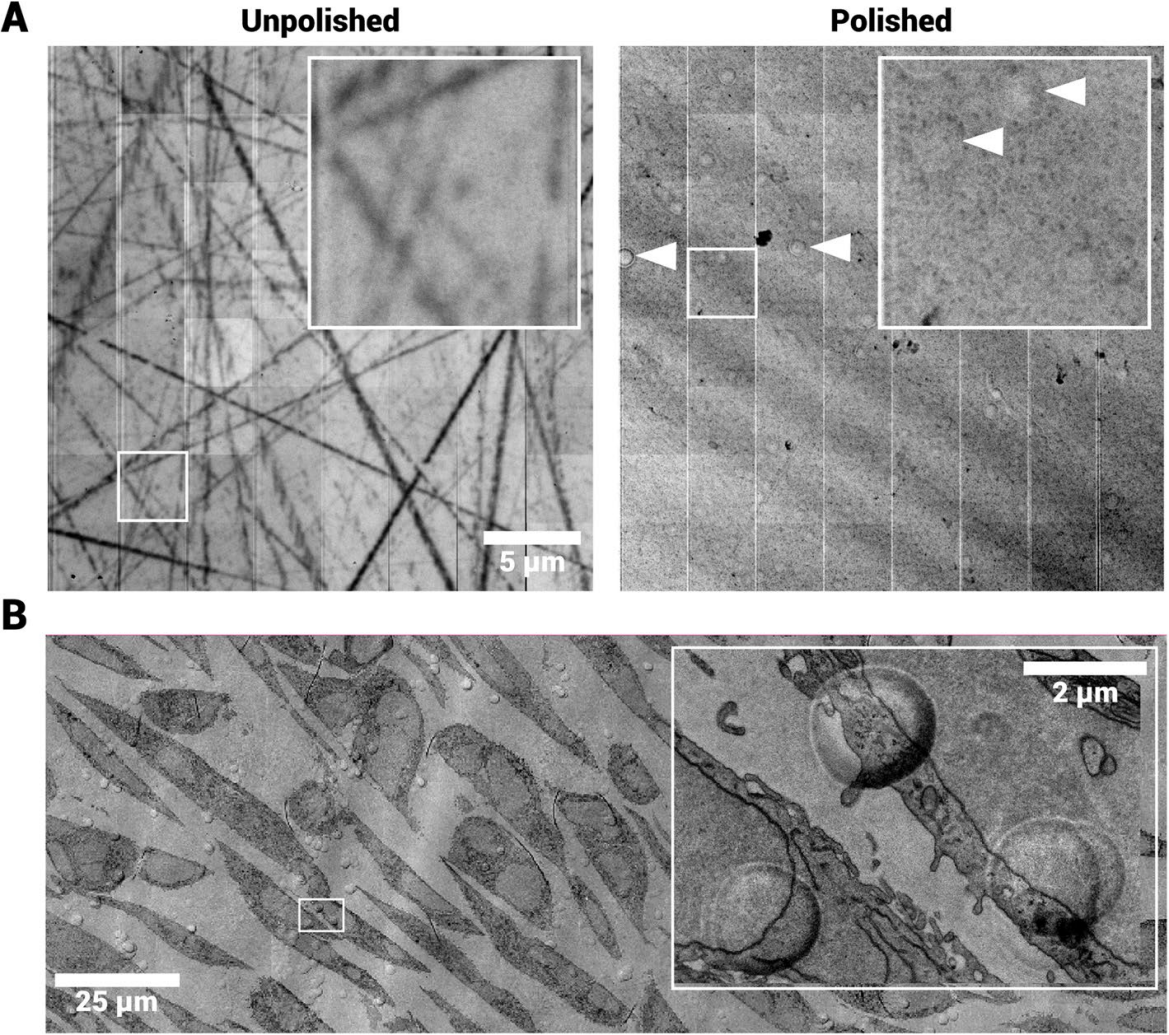
Effect of broad-ion beam polishing on substrate surface. **A** Single field in FAST-EM of both unpolished and ion-beam polished side of scintillator, coated on both sides with 30nm molybdenum. The unpolished side shows significant defects in the form of scratches. Arrows: dimple-like artifacts on the ion-beam polished side which are understood to be a result of the ion-beam polishing. **B** Milling artifacts visualized in FAST-EM dataset of cell culture, visible through the tissue section

The Ce:YAG substrates are by default mechanically polished after the growth process, though this may not be sufficient for imaging with OSTEM. Broad-ion beam etching further reduces surface roughness to a tolerable level for imaging. Without ion beam polishing, the (molybdenum-coated) scintillator surface contains many micrometer- and nanometer-scale defects (Fig. 6A, left panel), mostly appearing as scratches. These defects are visible in OSTEM images of ultrathin tissue sections (data not shown). Broad-ion beam milling removes these large defects, but the surface after milling shows apparent dimple-shaped structures of typically several micrometers in size (Fig. 6A, right panel).

The same artifacts are also observed in multibeam OSTEM datasets (Fig. 6B), in the sample and on the substrate surface. Here, the number of milling artifacts is particularly high, although usually their occurrence is rare. The ultrastructure can still be discerned through artifacts, but it is obscured at the edges, which is likely because of the increased curvature. A cross section of the substrate surface, made with focused ion-beam SEM (Fig. S3), shows elevations in the substrate of several hundreds of nanometers. The molybdenum film can be clearly discerned over the full field-of-view of the artifact. This observation is consistent with the fact that the transmission signal is not blocked in OSTEM detection (Fig. 6B). The artifacts appear to have a certain directionality as the transmission signal is most obscured on one specific side.

A subset of single-beam and multibeam OSTEM (FAST-EM) datasets shows large-scale, quasi-periodic intensity variations (Fig. 6B) reminiscent of ripples or waves, that arise from variations in dopant ion concentration (Zuidema 2023). They are commonly referred to as growth striations and are thought to form mainly by convection and temperature fluctuations in the growth interface (Zhu et al. 2017). The variations are unidirectional in the scintillator, possibly reflecting the growth direction of the crystal. In FAST-EM acquisitions, the artifacts have the same directionality in images from serial sections due to the fixed scan orientation. Interestingly, the intensity variations are only apparent on the broad-ion beam polished side of the scintillator, and “SPL” scintillators did not show these intensity variations. Taken together, though dimples and scintillation irregularities affect the resulting image, their occurrence is typically rare and homogeneous such that the interpretation of the ultrastructure is negligibly affected.

### Repeated scanning causes intensity differences in transmission imaging

**Fig. 7:**
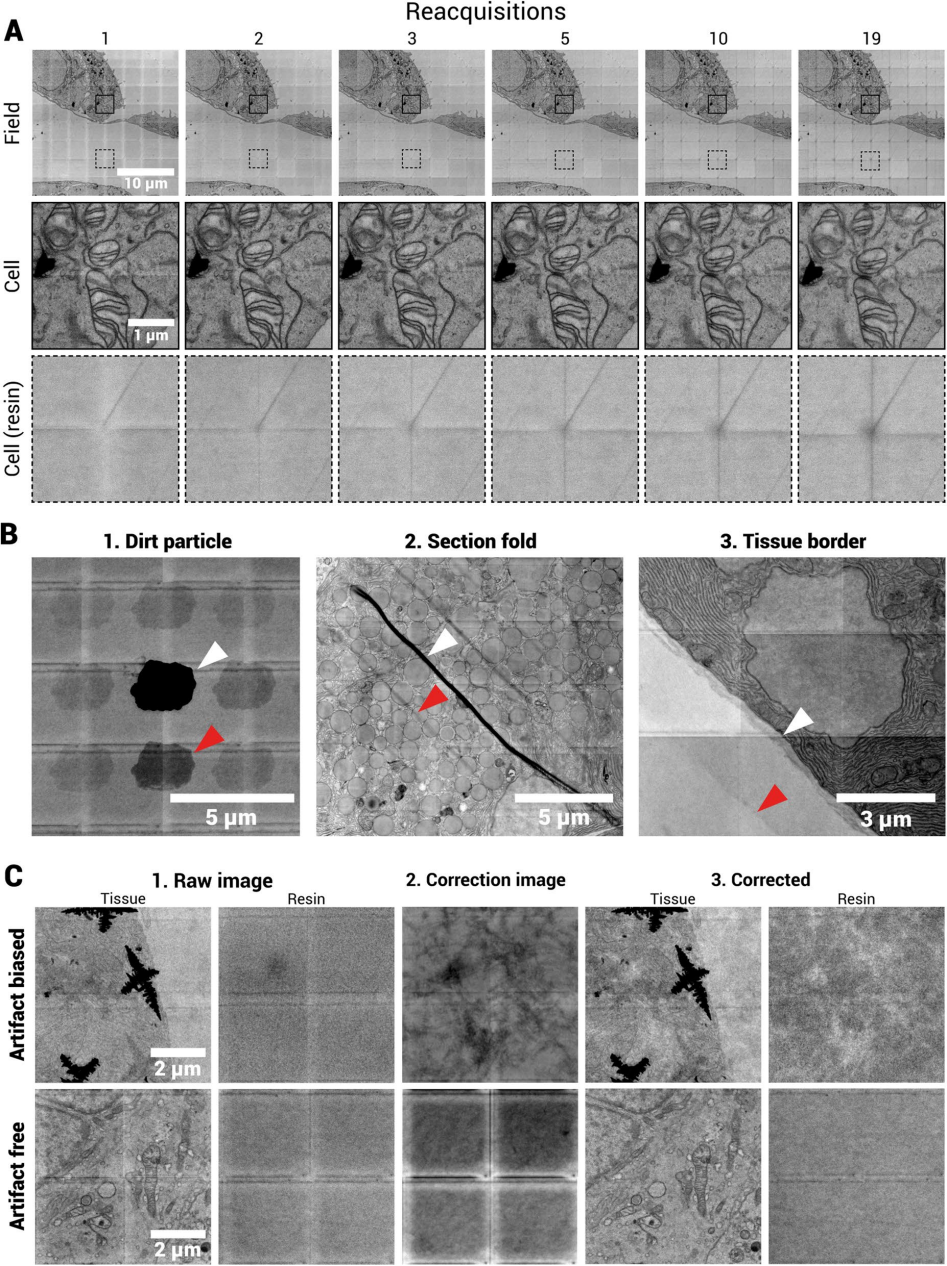
Mulitbeam OSTEM artifacts. **A** Beam damage artifacts caused by repeated acquisition with FAST-EM. The same field and cell images are shown through progressive scans of a MCF-7 cell sample. The full dataset can be viewed through Nanotomy.org. **B** Signal spillover creates “phantoms” in FAST-EM images. Images show several examples of high contrast features, including (1) a dirt particle, (2) section fold artifact and (3) a transition from tissue to empty resin. White arrows indicate the feature, red arrows indicate the phantom image of the feature in the neighboring cell. **C** Image artifacts generated from post-correction in a dataset with many artifacts (“Artifact biased”) and without artifacts (“Artifact free”). A 2x2 cell crop of a single field of tissue and empty resin is shown before and after post-correction, as well as the (average) correction image

In tiled acquisitions, some regions of the specimen are scanned repeatedly for overlap. In BSD, SE and STEM detection modes this can lead to contrast differences in the image due to thinning or carbon deposition. Similarly, in multibeam OSTEM, certain areas of the sample are scanned multiple times due to scan overlap between adjacent beams. Upon reacquisition of the same area (i.e. in case of acquisition errors), there is a cumulative effect, which becomes apparent in repetitive rescans with multibeam OSTEM from the same region-of-interest (Fig. 7A).

The first scan of the sample shows variations in intensity due to double or quadruple dosing and motivates the use of post-correction. Most notably, the overlapping scan areas of the adjacent beamlets show a higher image intensity (Fig. 7A, “Field” images), which could be explained by shrinkage or mass loss of the resin section under electron beam irradiation (Kizilyaprak et al. 2015; Skoupy et al. 2019), increasing the transmission coefficient. In the second and third scan, the intensity differences diminish, because the differences in section thickness are reduced by the dose accumulated from multiple exposures. From the 5th scan onwards, the effect of electron beam damage becomes increasingly apparent as horizontal, vertical and diagonal lines form on the sample (Fig. 7A, “Cell” and "Resin" images). The effect is most pronounced on the corners of the cell images, where the sample is exposed four times in a single scan (Fig. 1C). The edges of the cell have a lower intensity than the surroundings for scans 5 and onwards, due to increased carbon deposition. In conclusion, multibeam OSTEM images are characterized by intensity differences in overlapping scan areas, which reduce with repetitive scans but are also accompanied with increased beam damage.

### Signal spillover between neighboring beamlets introduces phantom artifacts

The optical system of the FAST-EM introduces some spillover between the signals of adjacent beamlets (Zuidema 2023). The interaction volume of the electron beam is converted and magnified by the scintillator and the optical detection system to a spot on the multipixel photon counter detector. The optical spot profile is a convolution of the electron beam interaction volume with the point spread function of the optical system, which will typically have tails since the interaction volume is not homogeneous. The optical spot profile (and hence the extent of signal spillover) is further influenced by aberrations, the contributions of which depend on the objective correction collar setting and the local scintillator thickness. The high NA objective is sensitive to variations in scintillator thickness or tilts of the scintillator on the holder.

The effect of signal spillover becomes most apparent when high contrast features are imaged, resulting in “phantoms” in neighboring cell images (Fig. 7B). It is apparent for certain artifacts caused by dirt particles or section folds, but also noticeable at sharp transitions in contrast from biological features. High contrast features attenuate the transmission signal and thereby the amount of spillover. The phantom artifacts are therefore thought to be a consequence of a reduction in the baseline spillover affecting neighboring detector signals due to relatively highly scattering areas.

### Post-correction of images introduces digital artifacts

The effect of artifacts can be aggravated by image processing. Post-corrected FAST-EM images sometimes show image artifacts which cannot be explained by the aforementioned mechanisms. Some datasets are corrupted by electron-dense artifacts (Fig. 7C, “Artifact biased” 1st column image). Image post-correction implemented in FAST-EM array tomography compensates for intensity differences caused by the multibeam scanning acquisition, by averaging all field images in a single region of acquisition and subtracting this average image from all other field images (3rd column in Fig. 7C, Kievits et al. (2024)). However, averaging represents all values and thus includes artifact-corrupted images. If the number of physical artifacts in the sample is sufficiently high, the post-correction image (3rd column) introduces pronounced digital artifacts, effectively adding an additional background texture to the post-corrected images (4th and 5th column in Fig. 7C). The effect is most pronounced in empty resin images that have been corrected (column 2 vs 5). For a dataset without prominent artifacts or electron-dense features (“Artifact free” in Fig. 7C), the correction image has a mostly uniform background, although intensity differences can still remain on the borders of cell images. The outlier removal procedure as described in Kievits et al. (2024) excludes fields with artifacts from the correction image, as long as the number of artifact-free fields in a single ROA remains above a threshold. Alternatively, the median image may be used as this is less affected by outliers.

### Effective mitigation of all artifacts

**Fig. 8:**
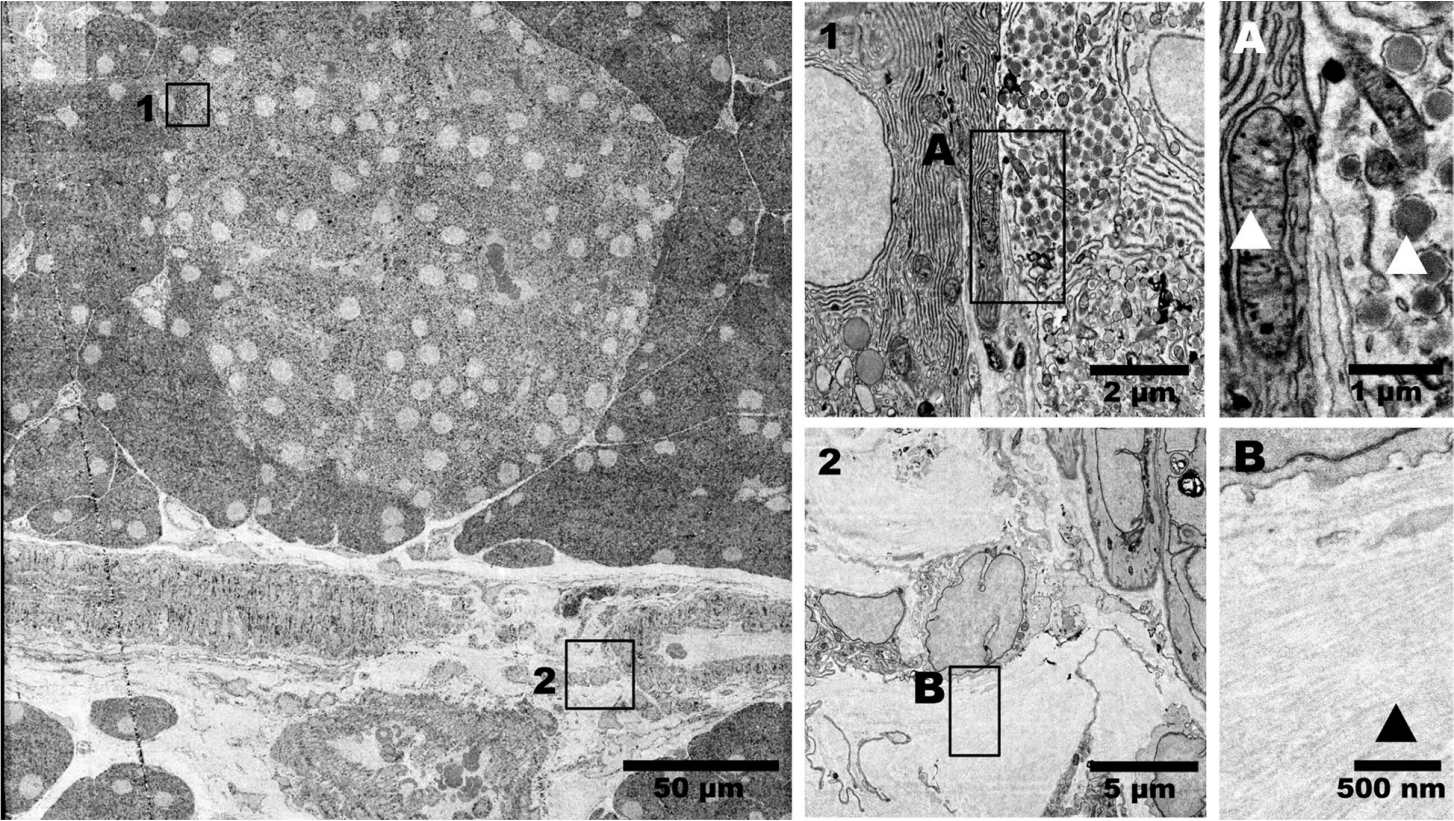
FAST-EM image quality with all artifacts mitigated. Image data represent a 250$\,\upmu$m x 250$\,\upmu$m acquisition of rat pancreas tissue, including Islet of Langerhans and surrounding exocrine tissue. Subpanels “A” and “B” show data at full resolution. Arrows in A indicate a mitochondrium with cristae and insulin granule, and in B collagen, respectively. Full dataset in can be accessed at Nanotomy.org

Lastly, mitigation strategies were applied to reduce or eliminate all aforementioned artifacts in subsequent acquisitions (Table 1). As an example, we include a FAST-EM dataset of rat pancreas acquired under optimized conditions (Fig. 8; data adapted from Duinkerken et al. (2025)). No significant artifacts related to sample preparation, substrate roughness, coating quality nor image processing are observed. Ultrastructural features such as mitochondrial cristae and vesicles are clearly resolved (Fig. 8A). In addition, the collagen-containing extracellular matrix is visible (Fig. 8B), indicating that the contrast of small biological features is now limited by the sensitivity of OSTEM detection and not by substrate artifacts. Collectively, these data demonstrate that OSTEM-related artifacts summarized in Table 1 are successfully mitigated under optimized acquisition conditions.

**Table 1:** Identified artifacts, their cause and mitigation strategies

Category	Type of artifact	(Possible) cause	Mitigation
Sample preparation	Post-staining artifacts	Heavy metal staining retention between the section and substrate	Use en bloc staining, perform washing steps of post-stained sections
	Electron-dense precipitates	Reaction between molybdenum coating and sample preparation chemicals	Use alternative coating (chromium), prepare sections with array tomography knife boat
Substrate quality	Reduced contrast, grainy images	Substrate roughness	Better surface polishing, perform quality control of substrates with AFM or white light interferometry
	Reduced tissue contrast, ultrastructure discernible	Low film coating quality	Quality control of substrates by visual inspection
	Milling artifacts	Broad-ion beam polishing	Substrate cleaning, quality check with SEM
	Directional intensity variations	Growth striations and fluctuations in cerium concentrations	Digital image correction, increase growth process stability, lower cerium concentration
Imaging, detection	Diagonal stripe patterns	Beam damage	Pre-exposure with defocused beam
	Phantoms	Spillover between adjacent beam signals	Minimize with optical objective correction collar
Image processing	Post-correction artifacts	Correction image biased towards electron-dense features	Correction with outlier exclusion or median image

## Discussion

OSTEM imaging is a relatively new approach that is well compatible with FAST-EM imaging (Fermie et al. 2021; Zuidema 2023; Kievits et al. 2024), but given the non-traditional sample preparation and specimen substrates, potential artifacts may be introduced which should be mitigated. OSTEM is incompatible with post-staining protocols because of apparent staining solution retention between the substrate and the specimen, leading to artifacts that obscure biological ultrastructure. This specific issue has not been reported in earlier array tomography studies where post-staining was applied to silicon substrates or ITO-coated coverslips, despite several studies using landing energies that would enable the electron beam to penetrate the underlying substrate for the used section thickness. Post-staining is typically applied after the sample is imaged with fluorescence microscopy (by means of immunolabelling or genetically encoded probes), because the fluorescence would otherwise be quenched by the heavy metal staining. Silicon wafers are typically cleaned and hydrophylized to improve section attachment by incubation in sulphuric acid and perhydrol (Horstmann et al. 2012) or by plasma treatment. Glass coverslips are gelatin-coated (subbed) to promote the adhesion of sections (Micheva and Smith 2007). However, it is unclear how thick the obtained gelatin layer is and whether a transmission signal can be recorded with OSTEM on subbed substrates. Post-staining artifacts can possibly be prevented by putting the sections onto a droplet of staining solution, then washing the sections and subsequently transferring them to the scintillator substrate with a loop. However, this only works for individual sections or small ribbons and is also more error-prone than on-substrate staining. A full conformal contact between the sections and the substrate may reduce or prevent these artifacts and they can be completely mitigated by pre-embedding staining.

The observed variability in appearance between coatings on different scintillators after specimen preparation were accompanied by contrast differences and artifacts in images of biological specimens, suggesting an affected coating. Electron precipitates formed on or below sections deposited onto a scintillator with a loop, may be formed by a reaction between the molybdenum coating and residues from the staining solutions (e.g. lead aspartate, thiocarbohydrazide). The absence of these artifacts when the scintillators is submersed in the knife boat, suggests that the water droplet facilitates the reaction between the coating and sample, whereas the large volume of water in the bath potentially dilutes the reaction product and prevents its formation at the sample-substrate interface. An alternative coating or an optimized sample and specimen preparation protocol can prevent the artifacts altogether. Therefore, the interaction of the coating layer with the specimen is not considered a general issue for OSTEM.

Oxidation of the molybdenum coating can explain the change in appearance and increased optical transparency after prolonged water or air exposure, since the reflectance of molybdenum oxide (0.14 at 550 nm) is less than metallic molybdenum (0.96 at 550 nm). Samples could be stored under inert gas to limit oxidation over prolonged time periods. For array tomography, especially the long-term stability of the film when immersed in the water bath is important. Alternatively, optimization of sputtering protocols and materials may yield coatings that are more resistant to air, water or chemical exposure, and with superior adhesion and reflectance, but the adhesion is linked to grain size which affects the surface roughness (Ahmed et al. 2020).

Irregularities in the substrate surface and doping concentration translate to contrast differences in FAST-EM data, which in turn reduce the biological contrast and impair the interpretation of biological ultrastructure. Absence of substrate artifacts in conventional array tomography datasets could be possible because the interaction volume of the beam is confined to the section at the used landing energies (Wacker et al. 2016; Peddie et al. 2017; Lane et al. 2022), or the beam does not get scattered by the underlying solid substrate (Micheva and Smith 2007; Burel et al. 2018). When the electron beam landing energy is increased, the substrate is revealed (Fig. 1A) and as a result the image quality deteriorates (Baatsen et al. 2021; Lane et al. 2021). To minimize substrate contribution to image contrast in OSTEM, we suggest that quality control of substrates prior to specimen preparation be performed by default.

Substrates with low surface roughness enable high-quality OSTEM imaging of biological samples. All measurements were performed with a single AFM tip, and the 2$\,\upmu$m FOV scans of “B” and “Delmic” scintillators, which were measured last, do not show the fine spatial features represented in the image of “SPL”. Furthermore, the measurements of these scintillators show a broadened central cross correlation peak. Both results may be explained by gradual AFM tip contamination by the biological specimen. The large roughness differences between “Delmic” and “B” scintillators are unlikely caused by tip contamination, implying that these must come from real substrate roughness variation. Since BSD images showed no apparent differences in image quality up till 3 keV landing energy (for higher energies the interaction volume extends into the scintillator substrate) the increased surface roughness explains the negative effect on the biological contrast as a function of the landing energy.

SEM inspection can yield a relatively quick assessment of relative surface roughness and suitability for OSTEM. Quantification of surface roughness by AFM or white light interferometry is a more objective way of assessing whether to pass or reject batches. Defining an unbiased passing criterium for scintillators based on the presented experimental data is difficult. The highest quality scintillators demonstrate RMS and mean roughness values below 1 nm and 2-3 nm peak height differences before specimen preparation. The coating of scintillator A and B, based on visual inspection, was clearly affected after biological specimen preparation and imaging, making it possible that the roughness before specimen preparation was lower. A minimum passing criterium could be defined as the average of scintillator A and “SPL”, which would roughly equate to 1.5 nm RMS/mean roughness and 10nm maximum peak height. However, the criterium may be less strict as the roughness can be underestimated by AFM tip contamination.

Whether to use a batch of scintillators for experiments can be further motivated by the presence of striations and milling artifacts. The extent of these artifacts may be determined prior to specimen preparation by imaging the substrate surface in single-beam or multibeam OSTEM. The structures are not composed of material redeposited on the surface by sputtering, as this type of artifact would be expected to attenuate the transmission signal. Instead, they could be caused by a decrease of the milling speed by contamination on the substrate. Since milling artifacts are relatively rare, they do not strictly pose a problem for image interpretation. Still, an extra cleaning step prior to broad-ion beam polishing, if not already part of the workflow, could reduce the occurrence of artifacts that obscure ultrastructure.

The observation that growth striation patterns are not seen in OSTEM datasets from “SPL” scintillators may be explained by differences in absolute cerium concentrations or tighter growth process control. Absolute cerium concentrations can be determined by X-ray diffraction analysis (Truc et al. 2025) and can guide optimal scintillator selection for OSTEM. A higher absolute cerium concentration in ’Delmic’ scintillators would explain their increased roughness, as cerium inclusion leads to defects in the crystal lattice. This would motivate the necessity of ion-beam polishing as well as the appearance of striations in OSTEM imaging, even though the latter may be obscured by other artifacts.

Signal spillover adds uncorrelated intensity contributions to multibeam OSTEM images, increasing the background intensity and reducing the effective image SNR and contrast. Thus, the amount of spillover in the system should be minimized, which should also reduce the effect of phantoms. The most effective way to achieve this, is by controlling aberrations with the objective lens correction collar. The PSF can be characterized by focusing a single beam on the substrate surface and acquiring a z stack on the diagnostic camera, while moving the objective lens through the optical focus position. The correction collar should then be set to the value that minimizes the point spread function tails.

The effect and extent of repeated electron-beam exposure and damage in multibeam OSTEM could be sample and embedding material specific. A thorough investigation on different samples and embedding materials was not performed, but may be interesting for future research, especially when extending FAST-EM to different applications. The ultrastructure remains discernable throughout repeated multibeam OSTEM acquisition, which shows that the sample is stable on the substrate. The content in the cell images does slightly change position in subsequent rescans, but this is also affected by the repositioning accuracy of the stage. In other transmission-based EM techniques such as serial-section TEM or large-scale STEM, ’prebaking’ or pre-exposure steps are typically performed at low magnification (Zheng et al. 2018; Scotuzzi et al. 2017). Primarily intended to reduce warping and shrinkage of the specimen under high-dose acquisition, such a procedure may also be useful to prevent e-beam induced artifacts in multibeam OSTEM. In TEM, a parallel beam is used to pre-irradiate the sample. Thus, pre-exposure in multibeam OSTEM should be performed with a defocused beam. Removing intensity differences induced by the multibeam scanning procedure would also eliminate the need for post-processing.

## Conclusions

OSTEM allows seamless imaging unobstructed by meshed grids, but the biological sample and solid substrate are in direct contact. The substrate condition may therefore affect the biological sample, or vice versa, leading to novel ways in which artifacts appear in EM images. We identified and investigated several artifacts when combining conventional EM sample preparation with scintillator substrates and OSTEM. It is shown that common artifacts in single and multibeam OSTEM can be attributed to interactions between the sample and scintillator substrate. Residual staining solution and coating-sample interaction give rise to artifacts which obscure biological ultrastructure. Furthermore, the quality of the substrate surface directly affects the image quality. A stable, homogeneous and resistant thin-film coating, as well as a high substrate quality and good surface polishing are prerequisites to mitigating potential artifacts and obtain high quality datasets. Furthermore, image interpretation can be complicated by the presence of broad-ion beam milling induced artifacts, striations, as well as phantom images created by signal spillover. Care should be taken during post-processing to prevent additional digital artifacts.

Awareness of artifacts in general helps discriminating biological features from artificial features. Understanding the root causes for how these artifacts arise, helps finding mitigation strategies and thus preventing their occurrence as demonstrated in this study.

## Materials & methods

All sample preparation and experiments were performed in the department of Imaging Physics at Delft University of Technology and the department of Biomedical Sciences, University Medical Center Groningen, Netherlands.

### Sample preparation

Zebrafish larva and rat pancreas samples were prepared as previously described (Kievits et al. 2024). Briefly, zebrafish larva were aldehyde fixed, subjected to reduced osmium post-fixation (1% osmium tetroxide and 1.5% potassium ferrocyanide in 0.1M sodium cacodylate buffer), *en bloc* stained with spun-down 4% neodymium acetate and dehydrated and flat embedded in EPON. Rat pancreas tissue was aldehyde fixed, vibratome sectioned, subjected to reduced osmium-thiocarbohydrazide-osmium (rOTO) post-fixation (1% osmium tetroxide, 1.5% potassium ferrocyanide and 4mM calcium chloride in 0.1M sodium cacodylate buffer (Holcomb et al. 2013)), *en bloc* stained with uranyl acetate followed by lead aspartate, dehydrated and flat embedded in EPON between ACLAR sheets.

### Specimen preparation

Thin-film coated (estimated 40 nm molybdenum), as well as uncoated yttrium aluminum garnet scintillator (ce:YAG) substrates were acquired from Delmic, Delft, Netherlands and Surface Preparation Laboratory, Wormerveer, Netherlands. For the uncoated scintillators, RF magnetron sputter coating was performed in-house with an AC450 (Alliance Concept, Annecy, France) operated at 150 W RF, 3 $\upmu$ bar for 32 s to achieve a layer of 30 nm molybdenum.

Ultrathin sections (80 or 100 nm) were cut using a UC7 ultramicrotome (Leica Microsystems, Wetzlar, Germany). For the rOTO stained rat pancreas sample, the scintillator substrates were submerged in a Ultra 35 Jumbo knife boat (Diatome Ltd, Nidau, Switzerland) before sectioning. After cutting ribbons, the water level was gently lowered to deposit the sections on the immersed substrate. For the other non-rOTO samples, ultrathin sections were cut using a Ultra 35 knife (Diatome Ltd, Nidau, Switzerland) and transferred to the scintillator using a Perfect Loop (Diatome Ltd, Nidau, Switzerland). No additional coating was performed before imaging.

### Electron microscopy

Backscattered electron detection (BSD) was performed in a Verios 460 SEM (FEI, Eindhoven, Netherlands) with the retractable concentric backscattered detector, employing a 1.5 keV to 4 keV landing energy, or in FAST-EM (Delmic, Delft, Netherlands) using the Trinity Detection System in-lens (T1) detector (typically 5 keV landing energy). SE detection was performed with the Everhart-Thornley detector in field-free mode or the through-the-lens detector in immersion mode, in the same Verios 460 SEM.

#### OSTEM

The setup for single-beam OSTEM is described in Kievits et al. (2024). Briefly, a SECOM integrated fluorescence microscope (Delmic, Delft, Netherlands) is retrofitted into a Verios 460 SEM with a fixed 6mm working distance. The emission filters of the fluorescence microscope are removed and the cMOS camera is replaced with a multi-pixel photon counter (Hamamatsu Photonics, Hamamatsu City, Shizuoka Prefecture, Japan). A 0.95NA air objective (Nikon Europe B.V., Amstelveen, Netherlands) is used for photon collection.

Large-scale OSTEM imaging was performed with the ODEMIS software (Delmic, Delft, Netherlands). The signal from the MPPC was rerouted into the SEM signal port of the SECOM hardware. We typically used a landing energy of 4keV, a dwell time of 5 $\upmu$s and pixel size of 4 nm (25900$\times$ magnification, 16.1$\,\upmu$m horizontal field width). The scan rotation angle was set in a way that the region-of-interest in the ultrathin section of interest was aligned with the FOV movement (a snake pattern, alternating movements right and left, downwards). Beam alignment, focusing and astigmatism correction were performed in the middle of the area of interest prior to acquisition. Next, a tiled acquisition was performed in ODEMIS with 10% overlap between neighboring tiles.

#### Large-scale imaging with FAST-EM

Large-scale datasets were acquired using the procedure as described earlier (Kievits et al. 2024). Briefly, samples were mounted on the FAST-EM holder and overview images were made using the backscattered electron detector. The electron beam was focused, corrected for astigmatism and FAST-EM specific calibrations were subsequently performed. All acquisitions were performed with a 5 keV beam energy, 0.4 nA beam current and 4 nm pixel resolution. Images from large-scale acquisitions were computationally stitched to generate a *mosaic* that covers the whole region of interest. The 2D reconstructions were rendered using Render^1^ and exported to a local instance of WebKnossos (Boergens et al. 2017) using custom Jupyter notebooks.

### Cross-section FIB

A cross-section was made with a Helios G3 DualBeam system (FEI, Eindhoven, Netherlands). A thin platinum layer was deposited on the sample by injecting a platinum precursor with the gas injection system and subsequent focused electron beam induced deposition. The sample was then tilted to 52 degrees to deposit more platinum using the focused ion beam deposition. A trench was milled with the FIB at 30 keV and with a 15 nA current. SE images were made in immersion mode using the through-the-lens detector (5 keV LE, 0.4 nA current and a 5 $\upmu$s dwell time).

### Light microscopy

Overview images of scintillators were acquired with a VHX-6000 digital microscope (Keyence Corporation, Osaka, Japan) at 100x magnification in epi-illumination mode, using the tiled-acquisition plugin.

### Atomic force microscopy

Atomic force microscopy of the scintillator substrates was performed with the Asylum Research Cypher system (Oxford Instruments, Abingdon, Oxfordshire, United Kingdom), using Fastscan-A cantilevers (Bruker, Billerica, Massachusetts, United States). The imaging mode used was AC (tapping) mode, with drive frequency of approximately 1.5 MHz and oscillation amplitudes of 5-10 nm. Images were background corrected in Gwyddion (Nečas and Klapetek 2012) using a polynomial subtraction (a=3), then row aligned using the median. Surface roughness values, measured as RMS roughness (Sq) and mean roughness (Sa), as well as height distributions were computed with the Statistical Quantities Tool after masking obvious dirt particles in the image with a simple height threshold and excluding these pixels from the calculation.

## Supplementary Information

Supplementary Material 1.

## Data Availability

The raw microscopy data underlying the figures is available through the 4TU repository (DOI: https://doi.org/10.4121/01efed58-9427-478a-8687-50b35daf3d9c). Access to large-scale datasets presented in this paper is provided through Nanotomy.org. Data in Figs. 6B and 8 was acquired prior to this study and reused for analysis.

## Abbreviations

EM: Electron Microscopy
SEM: Scanning Electron Microscopy
vEM: Volume Electron Microscopy
OSTEM: Optical Scanning Transmission Electron Microscopy
SE: Secondary Electron Detection
BSD: Backscattered Electron Detection
AFM: Atomic Force Microscopy

## Footnotes

1 https://github.com/saalfeldlab/render.git
