# Supplementary material for: Mitigation of artifacts in imaging biosamples with optical scanning transmission electron microscopy
Source: Appl Microsc. 2026 Apr 21;56:11. doi: 10.1186/s42649-026-00132-y (PMC13100101; doi:10.1186/s42649-026-00132-y)
Supplement: Supplementary file 1 — Supplementary Material 1. [file 42649_2026_132_MOESM1_ESM.pdf]

Supplementary figures

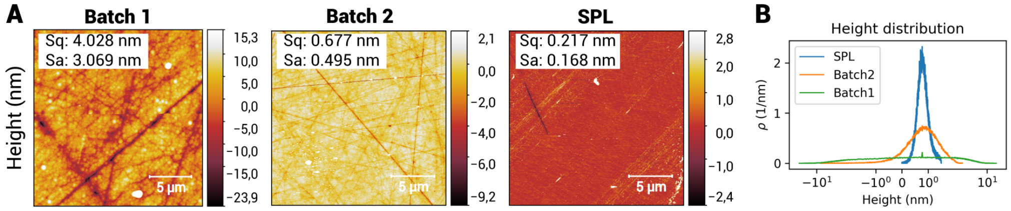

Figure S1: **A**: AFM measurements (20 µ m field-of-view) of uncoated YAG scintillators from different batches and suppliers show varying roughness and surface quality finish. Surface roughness is measured as RMS roughness (Sq) and mean roughness (Sa). **B**: Height distribution of images in **A**. Scintillators from batch 1 and 2 demonstrate numerous surface defects such as scratches, whereas the SPL scintillator is free of large defects.

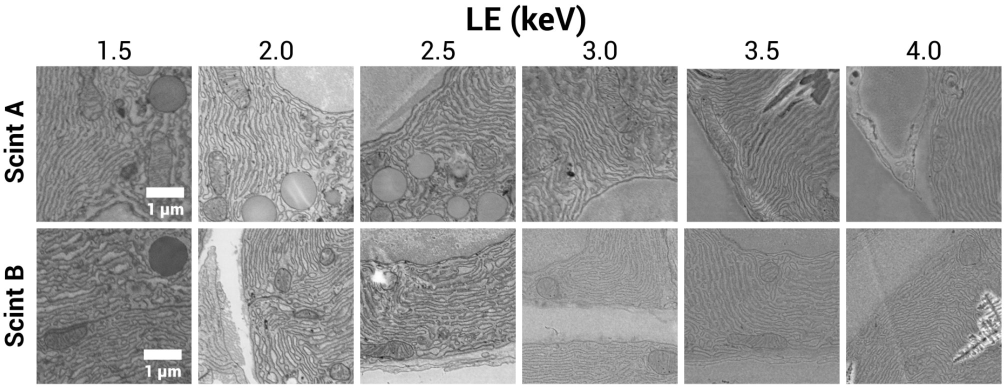

Figure S2: Biological image contrast in backscattered electron detection (BSD) as a function of electron landing energy (LE) for PD02 and PD11 scintillators. The image contrast is apparently similar for lower landing energies, but degrades more quickly for PD11 upon increasing the landing energy.

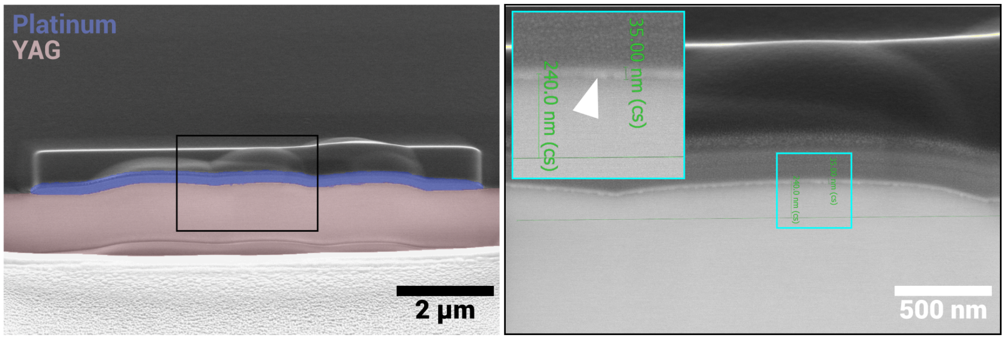

Figure S3: Focused ion beam cross section of a milling artifact. Indicated in blue and pink are the platinum protection layer and substrate (YAG), respectively. The black inset shows a zoom in on the intersection boundary of the platinum layer and substrate. In immersion mode, the molybdenum layer can be distinguished. The green lines indicate the distance from the top of the artifact to a line approximately parallel to the substrate surface next to the artifact, showing an elevation of approximately 240 nanometers, and a thickness estimate of the molybdenum layer respectively (white arrow in cyan inset).
